# Extensive Four-Dimensional Chaos in a Mesoscopic Model of the Electroencephalogram

**DOI:** 10.1186/s13408-015-0028-3

**Published:** 2015-08-12

**Authors:** Mathew P. Dafilis, Federico Frascoli, Peter J. Cadusch, David T. J. Liley

**Affiliations:** Department of Biomedical and Health Sciences, School of Health Sciences, Faculty of Health, Arts and Design, Swinburne University of Technology, PO BOX 218, Hawthorn, 3122 Australia; Department of Mathematics, Faculty of Science, Engineering and Technology, Swinburne University of Technology, PO BOX 218, Hawthorn, 3122 Australia; Department of Physics and Astronomy, Faculty of Science, Engineering and Technology, Swinburne University of Technology, PO BOX 218, Hawthorn, 3122 Australia; Brain and Psychological Sciences Research Centre, Faculty of Health, Arts and Design, Swinburne University of Technology, PO BOX 218, Hawthorn, 3122 Australia

**Keywords:** Chaos, Bifurcations, Brain dynamics, EEG

## Abstract

**Background:**

In a previous work (Dafilis et al. in Chaos 23(2):023111, 2013), evidence was presented for four-dimensional chaos in Liley’s mesoscopic model of the electroencephalogram. The study was limited to one parameter set of the model equations.

**Findings:**

In this report we expand that result by presenting evidence for the extension of four-dimensional chaotic behavior to a large area of the biologically admissible parameter space. A two-parameter bifurcation analysis highlights the complexity of the dynamical landscape involved in the creation of such chaos.

**Conclusions:**

The extensive presence of high-order chaos in a well-established physiological model of electrorhythmogenesis further emphasizes the applicability and relevance of mean field mesoscopic models in the description of brain activity at theoretical, experimental, and clinical levels.

## Findings

### Background

Chaos that requires at minimum four degrees of freedom to be adequately described is known as “four-dimensional chaos” (FDC). Lorenz [2] was one of the first to describe this phenomenon in 1984, and his results were given a firm numerical underpinning by Sigeti [3]. Other examples of such behavior exist in the literature but are generally rarely reported [4, 5].

Despite the complexity of human cognition and behavior, the focus on nonlinearity and chaos to date in neuroscience has been on low-dimensional phenomena. Freeman [6] provides a rare exception to this, describing a hierarchy of attractors, from point attractor dynamics under deep anaesthesia, to chaos in perception. Nonetheless, the importance of attractor dynamics in cortical activity is well documented, with significant implications for the brain at rest and while performing tasks [7–10].

Previous work shows evidence for FDC in Liley’s mesoscopic model of the electroencephalogram, which is related to an inverse period doubling cascade [1]. That cascade also accounts for intermittent behavior, which is reminiscent of burst-suppression-like phenomena occurring in anaesthesia [11] and epileptic encephalopathies [12]. In this report, we extend those findings and reflect on the importance of high-dimensional chaos in mathematical neuroscience models.

### The Liley Model

Liley’s theory of neural dynamics is a spatiotemporal theory of the electroencephalogram (EEG). Its mesoscopic character implies that it does not focus on fine neuronal detail, instead concerning itself with the dynamics of populations of neurons. The macrocolumnar formulation of the model consists of ten first-order coupled nonlinear ordinary differential equations parameterized by a significant number of physiological constants, which describe the neural population properties in detail. For further discussion of this formulation and a complete derivation, see [1, 13–15].

### A Shortcut to Extensive Four-Dimensional Chaos Search

We assume that the *n* Lyapunov exponents of an *n*-dimensional dynamical system described by ordinary differential equations (ODE) are ordered and given by $\lambda_{1}\geq\lambda_{2}\geq\cdots\geq\lambda_{n-1}\geq\lambda_{n}$. The largest integer *D* for which $\lambda_{1}+\lambda_{2}+\cdots+\lambda_{D}\geq 0$ is called the *topological dimension* of the attractor [16]. The next highest dimension ($D+1$) is the minimum integer dimension is which the attractor can exist.

Previously [1], the parameter set investigated provided the following first four *λ* values (base *e*, per second): $\lambda_{1}=9.6$, $\lambda_{2}=0$, $\lambda_{3}=-6.4$, and $\lambda_{4}=-11.5$. This means that $\lambda_{1}+\lambda_{2}+\lambda_{3} = 3.2$, and $\lambda_{1}+\lambda_{2}+\lambda_{3}+\lambda_{4}=-8.3$. So, in that case $D=3$ and $D+1 = 4$, and the minimum integer dimension in which the attractor can exist is four: this is why the claim of FDC holds. It is important to realize the difference between the so-called hyper chaos, which has been studied extensively in maps and ODEs, and FDC. In FDC the topological dimension is at least three as in the case of hyper chaos, but FDC is characterized by only a single positive Lyapunov exponent, whereas hyper chaos is associated with two or more positive exponents (i.e. $\lambda_{1}>0$ and $\lambda_{2} > 0$).

Building on the insights from previous work, we here look for cases where ${\lambda_{1} > \lvert\lambda_{3}\rvert}$ and $\lambda_{2}=0$. Since the exponents are ordered, we have $\lambda_{1}+\lambda_{2}+\lambda_{3}>0$ and $\lambda_{1} + \lambda_{2} + \lambda_{3}+\lambda_{4}<0$, with $\lambda_{3}<0$ and $\lambda_{4}<0$. As such, *D* will always be at least three, and the minimum integer dimension that the attractor can exist in is at least four. Also note that the Kaplan–Yorke (or Lyapunov) dimension $D_{\mathrm{KY}}$ [16] is given by

1 $$ D_{\mathrm{KY}}=3-\frac{\lambda_{1}+\lambda_{2}+\lambda_{3}}{\lambda_{4}}, $$

hence, with the above *λ*’s, $D_{\mathrm{KY}} > 3$ always. In other words, for the purpose of our investigation it here suffices to examine the largest three Lyapunov exponents, dramatically decreasing the computational burden associated with the search. It is in fact possible to state that the chaos is at least four-dimensional, if $\lambda_{1} > \lvert\lambda_{3}\rvert$ when $\lambda_{1}>0$ and $\lambda_{2}=0$, without evaluating the full spectrum of ten exponents. This reduces the original computation from 120 coupled nonlinear ODEs to only 43. The calculation for the present report has been performed using the well-known Christiansen–Rugh algorithm for the partial Lyapunov spectrum [17], under the same boundary conditions and simulation lengths as discussed in previous studies [14].

### Extension of High-Order Chaotic Dynamics in the Liley Model

It is convenient to study the extension of the FDC respect to two important model parameters, $p_{ee}$ and $p_{ei}$, which are the excitatory and inhibitory input pulse densities to the modeled excitatory neural population. These are expected to vary most considerably and widely physiologically, capturing the effect of incoming thalamo-cortical input. The other physiological parameters of the model are the same as in Ref. [1].

Investigation of 433,316 different $p_{ee}$ and $p_{ei}$ combinations, selected uniformly at random from a biologically relevant section of the $p_{ee}$–$p_{ei}$ plane (i.e. $0< p_{ee}\leq 30$, $0< p_{ei}\leq 10$), has been carried out. Selecting sets that show the largest Lyapunov exponent (LLE) being positive, 158,013 of the total points showed a $\mathrm{LLE}\geq 1$ per second, base *e*. We use this threshold to avoid ambiguity with exponents that have a slow convergence to zero. Out of these clearly chaotic sets, 34,533 or 21.8 % of the chaotic instantiation of the model had $\lambda_{1} > \lvert\lambda_{3}\rvert$, exhibiting FDC, i.e. about 8 % of the overall points that have been simulated.

**Fig. 1:**
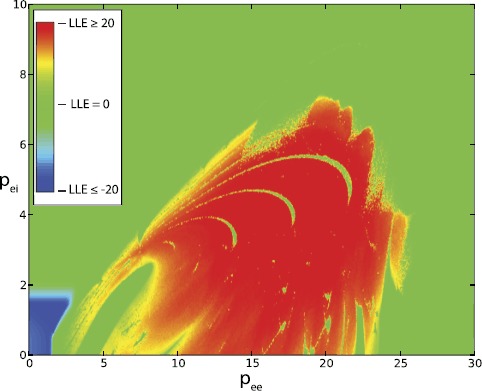
Largest Lyapunov exponent values (LLE) plotted as a function of $p_{ee}$ and $p_{ei}$. *Blue* is for $\mathrm{LLE} \leq -20$, *green* is for $\mathrm{LLE} \approx 0$, and *red* is for $\mathrm{LLE}\geq 20$, all values per second, base *e*. Thalamic inputs are expressed in units of ms^−1^

Figure 1 illustrates the behavior of the LLEs of the system as a function of $p_{ee}$ and $p_{ei}$. The color scale is such that blue represents an $\mathrm{LLE} \leq -20$, red means $\mathrm{LLE} \geq 20$ and green corresponds to a value of approximately zero. Again, this is a consequence of the slower rates of convergence for exponents associated with periodic orbit dynamics, which do not always correspond to exactly zero at the end of the simulation run. The extensive nature of chaos in this plane is evident, as are the limit cycles ($\mathrm{LLE} = 0$, green) and point attractors (negative LLE, blue). Note also the periodic windows interspersed among the chaotic fringes.

**Fig. 2:**
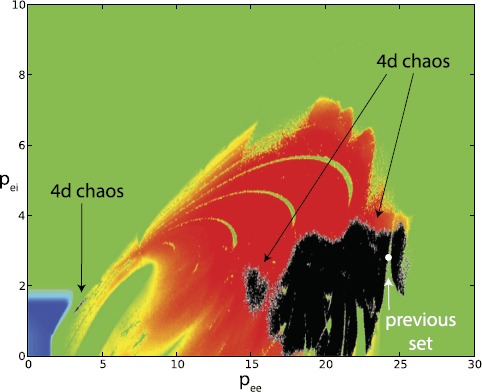
LLE plotted as a function of $p_{ee}$ and $p_{ei}$, with four-dimensional chaotic points in *black*. The parameter set discussed in our previous work [1] is indicated by *a white circle* and corresponds to $p_{ee}=24.2453\mbox{ ms}^{-1}$, $p_{ei}=2.299\mbox{ ms}^{-1}$

Superimposing the high-order chaotic points (in black), Fig. 2 gives an illustration of the extension of the FDC region. The overall set of FDC points is made of three different areas: one very extended and of irregular shape at large values of $p_{ee}$, one limited and more regularly shaped at intermediate values of $p_{ee}$ and a very small, elongated collection of points at low $p_{ee}$, $p_{ei}$, far from the chaotic (red) region and bordering with the sea of periodic activity (in green). This region is particularly interesting, since it corresponds to small values of the thalamic input, which are associated with ordinary thalamic activity. In our previous work, instead, we reported FDC for a parameter set with a high value of $p_{ee}$, possibly corresponding to a pathological or other abnormal state.

**Fig. 3:**
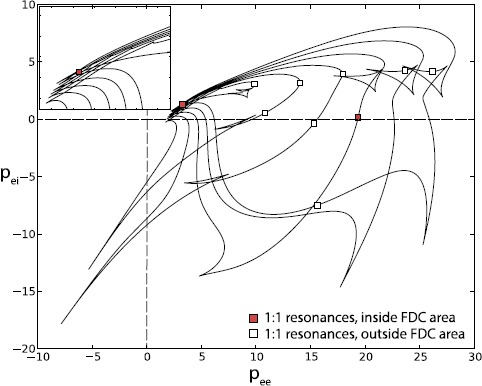
Continuation of codimension-two points associated with SNICs in the full $p_{ee}$–$p_{ei}$ plane, i.e. not limited to biologically relevant values. *The inset* shows the accumulation of SNICs lines in the area of high-order chaos at low values of $p_{ee}$, $p_{ei}$. Strong resonance occurring inside the four-dimensional chaotic set are shown as *red squares*. Other 1:1 resonances are shown as *white squares*

**Fig. 4:**
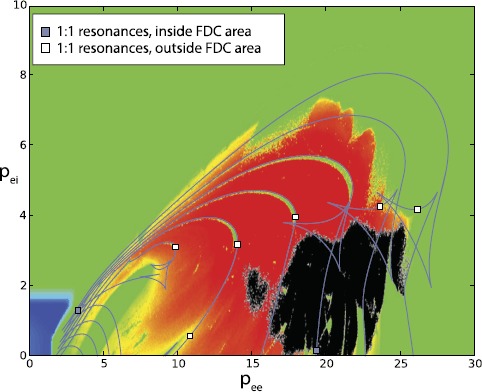
Overlapping of SNICs lines (*light blue*) with LLE plotted as a function of $p_{ee}$ and $p_{ei}$. *Points in black* are where the chaos is four-dimensional. The strong resonances inside the chaotic areas (in the vicinity of $(3.5, 1.5)$ and $(19.5, 0.2)$) are now indicated by *light blue squares*

Using the bifurcation package AUTO [18], a continuation in the two model parameters for the saddle-node on invariant cycle (SNIC) points of the periodic orbits allows for some speculation on the genesis of such intriguing, high-order phenomena. Figures 3 and 4 show a partial and full analysis, in conjunction with the plot of LLEs. Two 1:1 resonance points [19], highlighted in red in Fig. 3 and light blue in Fig. 4, overlap with sections of the FDC area. Lines of SNICs also correspond to the finger-shaped gaps (in green) for $\mathrm{LLE} \approx 0$. Further strong resonances often occur at the end of such gaps. The complex bifurcation scenario that may arise from such strong resonances could be partially responsible for the FDC, in particular for the high-order chaos occurring at low $p_{ee}$ values. Generation of FDC has also been associated with an inverse period doubling cascade in our previous work, and it could be possible that similar mechanisms control FDC for high $p_{ee}$ values. A thorough analysis of the processes involved is beyond the scope of this report.

### Conclusions

The results presented here show the extent of four-dimensional chaos within a biologically relevant parameter slice of Liley’s model. The fact that FDC is so extensive suggests extreme caution when performing visual inspections or even nonlinear time-series analyses to match experimental EEG traces with brain states or functions [20, 21]. In fact, the chaotic attractor associated with FDC dynamics for a specific parameter set at high $p_{ee}$ shows an amorphous appearance [1], which may make it hard to distinguish from noise. Combinations of noise and cortical activity already appear to be very difficult to untangle for dynamics simpler than the one discussed here [22].

A novel, important finding is that high-dimensional chaos is not limited to pathological or abnormal brain states but is present also for values of the thalamic input well inside the ordinary range of thalamic activity, i.e. $0 < p_{ee} < 10\mbox{ ms}^{-1}$. Hence, activity associated with high-dimensional strange attractors could occur more frequently than so far assumed. This aspect has also relevance as regards multistable behavior, given that Liley’s model can support multistable dynamics induced by different attractors [15]. Multistability has in fact been shown to capture aspects of brain activity in a variety of important neurological settings, including perceptual decision making and critical behavior of the brain at rest [23–25]. We hope that our findings may inspire further research work into the role of high-order chaotic dynamics in brain activity.
